# Exhaled carbon monoxide: variations due to collection method and physiology

**DOI:** 10.1088/1752-7163/adba05

**Published:** 2025-03-14

**Authors:** Shahriar Arbabi, Eric P Smith, Jacob J Fondriest, Nagako Akeno, Robert S Franco, Robert M Cohen

**Affiliations:** 1 Department of Internal Medicine, Division of Endocrinology Diabetes and Metabolism, Cincinnati College of Medicine and Cincinnati Veterans Affairs Medical Center, Cincinnati, OH, United States of America; 2 Division of Pathology and Laboratory Medicine, Cincinnati Children’s Hospital Medical Center, Cincinnati, OH, United States of America; 3 Department of Internal Medicine, Division of Hematology-Oncology, University of Cincinnati College of Medicine, Cincinnati, OH, United States of America

**Keywords:** alveolar carbon monoxide, end-tidal carbon monoxide, hemolysis, carbon monoxide, red cell lifespan

## Abstract

The measurement of exhaled carbon monoxide (eCO) is relevant to understanding normal physiology and disease states but has been limited by deficiencies in valid sampling protocols, accurate and feasible measurement methods, and the understanding of normal physiological variation. The purposes of this study were (**1**) to compare the three collection methods for eCO and (**2**) to gain a better understanding of patterns of normal variation by obtaining repeated daily and weekly measurements. We compared three techniques to sample eCO: continuous breathing **(ConB)**, breath-holding **(BrH)**, and short rebreathing (**SrB**). We used a Carbolyzer mBA-2000 instrument that involves an electrochemical method to quantify CO, with the final value corrected for ambient CO. In **Phase I**, we compared **ConB** with **BrH** in 10 healthy non-smokers (5 male, five female). On day 1, the eCO was determined from 07:30 to 17:00 (11 samples), and the first four morning time points were repeated on days 7, 14, and 21. **ConB** had a lower eCO than **BrH,** and eCO_2_ was frequently below the threshold of 4.6% compatible with inadequate alveolar sampling. The eCO measured by the **ConB** and **BrH** methods increased during the day and showed week-to-week variability. In **Phase II**, we compared the **BrH** and **SrB** techniques by collecting prebreakfast samples weekly for four weeks in 30 healthy non-smokers (15 male,15 female). Comparing the **SrB** vs. the **BrH** method, **SrB** was the easier for the participants to perform, generated higher eCO (∼ 0.5 ppm), and produced higher eCO2 levels (5.2% ± 0.3 vs. 5.0% ± 0.2); Importantly, **Phase II** study revealed that week-to-week changes in prebreakfast fasting eCO for individual participants were ⩾1.0 ppm in ∼ 37%. This variability complicates the interpretation of the relationship between small changes in eCO and the underlying physiological or disease states.

## 1. Introduction

In 400 BC, Hippocrates described the detection of odors on human breath, marking one of the earliest documented instances of ‘analytical’ health assessment [1]. Today, the constituents of breath currently considered to have potential relevance to disease fall into three categories: (1) organic and inorganic gases, (2) volatile compounds, and (3) condensates with aerosol particles [2]. The focus of this study was the measurement and interpretation of exhaled carbon monoxide(eCO) and how this relates to the use of eCO to assess health and disease [3].

Endogenous CO arises from the breakdown of heme proteins, primarily hemoglobin (75%–80%) and other hemoproteins such as myoglobin, cytochromes, peroxidases, and catalase [3]. Heme oxygenase (HO) enzymes (HO-1, HO-2, and HO-3) regulate this degradation process, producing biliverdin/bilirubin, ferrous ions, and CO [4]. Although these enzymes are found in most tissues, the reticuloendothelial system of the liver and spleen is the primary site of heme catabolism, and consequently, the major producers of endogenous CO [5–8].

While endogenous CO can be converted to CO_2_ by cytochrome C oxidase in the mitochondria, its primary elimination from the body occurs through exhalation, i.e., diffusion across the alveolar-capillary membrane [9, 10]. Therefore, assuming negligible exhaled CO originating from the environment [11], the concentration of eCO mirrors the endogenous carbon monoxide production. However, although other sources of CO are low, this tissue-specific HO enzyme activity can modulate localized CO cell signaling functions [4, 12], and in some disease states, such as asthma, leads to increased systemic and exhaled CO [13].

Much of the challenge in the interpretation of eCO, independent of source considerations, relates to the impact of the methods of collection and detection. Many original studies [14] in the 1950s and the 60’s focused on labor-intensive and complex strategies in which the rate of endogenous production of CO (or blood COHb) was measured in a closed system [15]. Subsequent efforts to develop more practical and feasible measures of eCO have yielded a variety of alternative collection methods, most commonly quantification of end-tidal CO (ETCO) that correlates well with CO production approaches [16, 17]. These more feasible and practical methods have generally combined simplified and standardized collection with less cumbersome CO detection technology in which issues of specificity and sensitivity are addressed [18, 19]. In most studies, eCO has been measured with less expensive and more portable electrochemical devices than with infrared or gas spectrometry methods. The enhanced accessibility of eCO measurements has led to an impressive effort to relate measured CO to numerous clinical conditions, including smoking cessation [20], asthma [21], hemolysis [22, 23], and inflammatory diseases [24].

In this study, we compared three different methods for collecting eCO: a breath-holding technique (**BrH**) similar in principle to that used by Levitt [17, 25] and two others involving ‘continuous breathing (**ConB**)’ and ‘short rebreathing’ (**SrB**)’ techniques [26, 27]. All three are suitable for routine use. As part of the comparison, we addressed aspects of eCO collection that could affect its interpretation. Specifically, we addressed the reproducibility of eCO over the course of the day and when repeated weekly over the course of four weeks. We found that (1) there was a significant diurnal eCO variation, (2) levels repeated weekly showed significant variation in a substantial subset of subjects, which was not explained by any intercurrent medical issues or changes in ambient environmental CO in the Clinical Research Unit (CRU), and (3) the **SrB** and **BrH** methods are likely to reflect end-tidal better than **ConB**. These results raise questions regarding the validity of a single eCO measurement in applications where small changes have a putative physiological meaning.

## 2. Methods

### 2.1. Participants and study design

**Figure 1.**
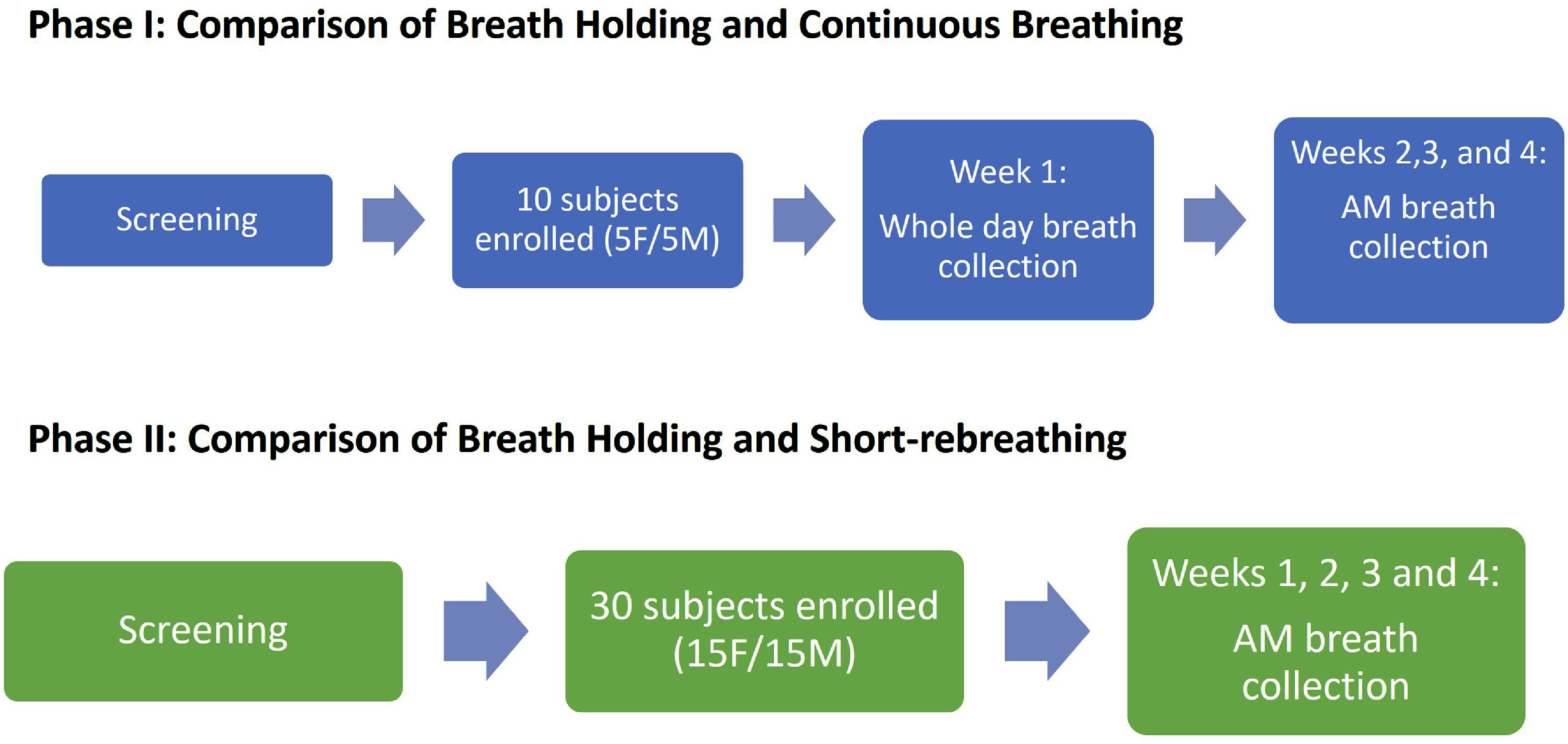
Flow charts of Phase I and Phase II of the study.

This study was conducted in two phases (figure 1) at the CRU of the Cincinnati VA Medical Center (CVAMC. After obtaining consent, we measured the height, weight, and resting vital signs. We collected blood and urine samples to establish baseline values and assessed the criteria outlined in table 1 for inclusion and exclusion.

**Table 1.** Study participants’ inclusion and exclusion criteria.

Inclusion	Medical history exclusion	Laboratory test exclusion
18–75 years old	**Hematologic:**	**Hematologic:**
Non-pregnant	Known hemoglobinopathy, red blood cell disorder, coagulopathy, or serum protein disorder such as multiple myeloma	Hemoglobin < 10 gm/dl; Reticulocyte count > 2%
Non-smoker	Blood donation or surgery within the preceding three months, history of GI blood loss, or recent hematoma	MCV: outside 80–100 femtoliters range WBC: outside 3800–10800 *μ*l^−1^ range
	Medications affecting heme-oxygenase activity	Platelet count outside 140–400 000 *μ*l^−1^ range
	**Pulmonary:**	**Renal:**
	Asthma, COPD, or cystic fibrosis	Spot urine microalbumin > 30 mcg mg ^−1^ creatinine
	Lung transplantation	
	**Other:**	**Hepatic:**
	Active infection	Transaminases > 3 X the upper limit of normal
	Known rheumatologic disease	
	Pregnant, nursing, or planning pregnancy	
	⩾ NYHA stage 3 heart failure	
	Irradiation	
	History of hypo- or hyperthyroidism	
	**Environmental Exposure:**	
	Open fire or wood stove at home	
	Job-related exposure to CO	
	Plans to move from area during study.	

MCV: mean corpuscular volume; WBC: white blood cell count.

**Phase I**

**Phase I** investigated the optimal time for eCO collection by repeated sampling throughout the day (before breakfast to before dinner) and repeated weekly prebreakfast sampling over 4 weeks. We compared two breath gas sampling methods, Continuous breathing (**ConB)** and breath holding (**BrH**). We enrolled ten healthy, non-smoking subjects aged 18–76 (5 males, 5 females) who met the criteria in table 1. Participants consumed a light, low-carbohydrate meal the night prior and fasted overnight. To reinforce the importance of exhaling slowly and steadily during eCO collection, the participants, prior to starting the study, were trained to exhale steadily into a filling bag with the goal of filling the bag over approximately 10 s. Before each collection session, they were then asked to sit quietly for approximately 30 min or until their vital signs approached baseline/resting values (within ± 10% resting vital signs at the time of enrollment visit). Breath sample collection commenced at 08:00, with samples collected every 30 min before breakfast (totaling four collections). Breakfast was provided at approximately 10:00, after which breath samples were collected every hour from 11:00 to 17:00. (11 CO measurements). For each time point, we obtained one sample using **ConB** and one sample using **BrH** and alternated the technique used first. There was at least a five-minute interval between the two collections. The subjects also returned weekly for three more weeks for four eCO measurements before breakfast using both **ConB** and **BrH** methods.

**Phase II**

**Phase I** results suggested that the **BrH** method was superior to **ConB** and that a fasting sample before breakfast was the more stable baseline for CO measurement. However, because of apparent significant week-to-week variation even in the prebreakfast fasting sample, **Phase II** assessed the week-to-week reproducibility of the morning breath sampling by expanding the number of subjects to 30 and substituting the short rebreathing (**SrB)** technique for the **ConB** method in addition to retaining the **Phase I** participants [28]. We invited all **Phase I** participants to enroll in **Phase II**. Four male and four female participants agreed to participate and provided consent for **Phase II**. Additionally, we enrolled 22 new participants, resulting in a total of 30 participants (15 males and 15 females). Participants were instructed to consume a light, low-carb meal the night before the measurement session, fast overnight, arrive at the research clinic at around 07:30, and sit quietly for approximately 30 min. Similar to Phase 1, the importance of slow and steady exhalation was emphasized for any new subjects and reinforced for those subjects previously enrolled in **Phase I**. Breath samples were collected before breakfast, starting at 08:00 and repeated every 30 min until 1000.

### 2.2. eCO and carbon dioxide measurement

The concentrations of CO and CO_2_ in the exhaled breath were measured using a Carbolyzer (Carbolyzer mBA-2000 TAIYO Instruments INC, Osaka, Japan) [29]. This instrument was equipped with a sensitive electrochemical detector for CO measurement with a range of 0–50 ppm and a sensitivity of ± 0.1 ppm. The Carbolyzer instrument measured the concentration of carbon dioxide (CO_2_) at the same time as CO. The CO_2_ concentration was used as an indicator that the collected gas represented accurate sampling of alveolar gas (CO_2_ ⩾ 4.62%) [30]. The analyzer was capable of continuous side-stream sampling with a flow rate of 0.2 l min^−1^. With the objective of minimizing ambient CO variation, the selected well-ventilated study room was monitored for CO levels over several weeks to confirm that the CO concentration remained stable between 100–200 PPB (0.1–0.2 PPM) when the room was unoccupied and inactive. Additionally, the potential influence of at least two people being present in the room was assessed and confirmed to not affect the measured CO levels.

The Carbolyzer continuously aspirated air from the sampling tube and displayed ambient baseline CO values. The CO value was adjusted by subtracting the ambient CO concentration measured by the Carbolyzer before each breath sample collection. The specificity of CO detection by the Carbolyzer is affected by cross-interference from high concentrations of H_2_ (1 ppm H_2_ = 0.0035 ppm CO) that can be present, in particular, after meals. Therefore, in **Phase I**, to correct for potential interference, the concentration of H_2_ in the same breath samples was analyzed using a QuinTron BreathTracker SC Analyzer (QuinTron, New Berlin, WI, USA). The eCO measured by the Carbolyzer was corrected for H_2_ by subtracting 0.0035 ppm for each detected ppm of H_2_. Because the **Phase I** results indicated insignificant levels of H_2_ in a fasting state, H_2_ concentrations were not measured in **Phase II** samples.

The Carbolyzer instrument was calibrated for the zero point and the sensitivity (span point) of the CO/CO_2_ detector. Calibration was performed at least once every week with three known mixtures: purified air without CO or CO_2_, 1.8 ppm CO and 5% CO_2_ balanced air, and 6 ppm CO and 10% CO_2_ balanced air (Praxair, Cincinnati, Ohio). In addition, to perform an assessment of the Carbolyzer precision before initiating the study, we collected eCO using the BrH technique on 12 individuals. The collection was repeated three times with a brief minute interval between each collection. This was repeated on a separate day. The average SD and CV for both days were 0.05 and 3%.

### 2.3. eCO collection techniques

#### 2.3.1. *Breath-holding technique (*figure 2*, Panel (B)):*

A nose clip was placed with the participant in a standing position and at rest. A deep breath was held for 30 s and then exhaled into the QuinTron AlveoSampler breath collection kit (QuinTron, New Berlin, WI), consisting of a mouthpiece, T-piece flutter valve, accessory discard bag, and sample bag. The exhaled gas first fills the accessory bag with dead-space gas, and then a valve opens, directing the alveolar expiratory gas to the sample bag. The sample bag was connected to the Carbolyzer machine to measure CO and CO_2_ concentrations. A CO_2_ concentration of 4.6% (equivalent to CO_2_ 35 mmHg) or higher was considered to indicate an adequate alveolar gas sample [27, 31, 32].

#### 2.3.2. *Continuous breathing technique (*figure 2*, Panel (A))*

A nose clip was placed with the participant in a standing position and at rest. Exhaled air was directed into a T-tube with a one-way flutter valve that allowed ambient room air to be inhaled but prevented mixing of exhaled gas with ambient air. A side port connected the collected gas directly to the Carbolyzer machine [31].

#### 2.3.3. *Short rebreathing technique: (*figure 2*, Panel (C))*

A closed breathing circuit was a modification of previously described approaches for measuring carbon monoxide lung diffusing capacity [33, 34]. The circuit was simplified to include a non-latex 3.0 l anesthesia bag with two valves and a face mask with a sealing cushion to fit securely on the participant’s face. At the beginning of the sampling, the anesthesia bag was filled with a mixture of room air and oxygen (4 ml kg^−1^ body weight, consisting of 2/3rd room air and 1/3 oxygen from the hospital clinic’s oxygen outlet) to minimize potential discomfort from rebreathing in a closed system with a low oxygen concentration. The anesthesia bag served two purposes: as a reservoir for the air/oxygen mixture and as a container for exhaled mixed alveolar gas during rebreathing.

**Figure 2.**
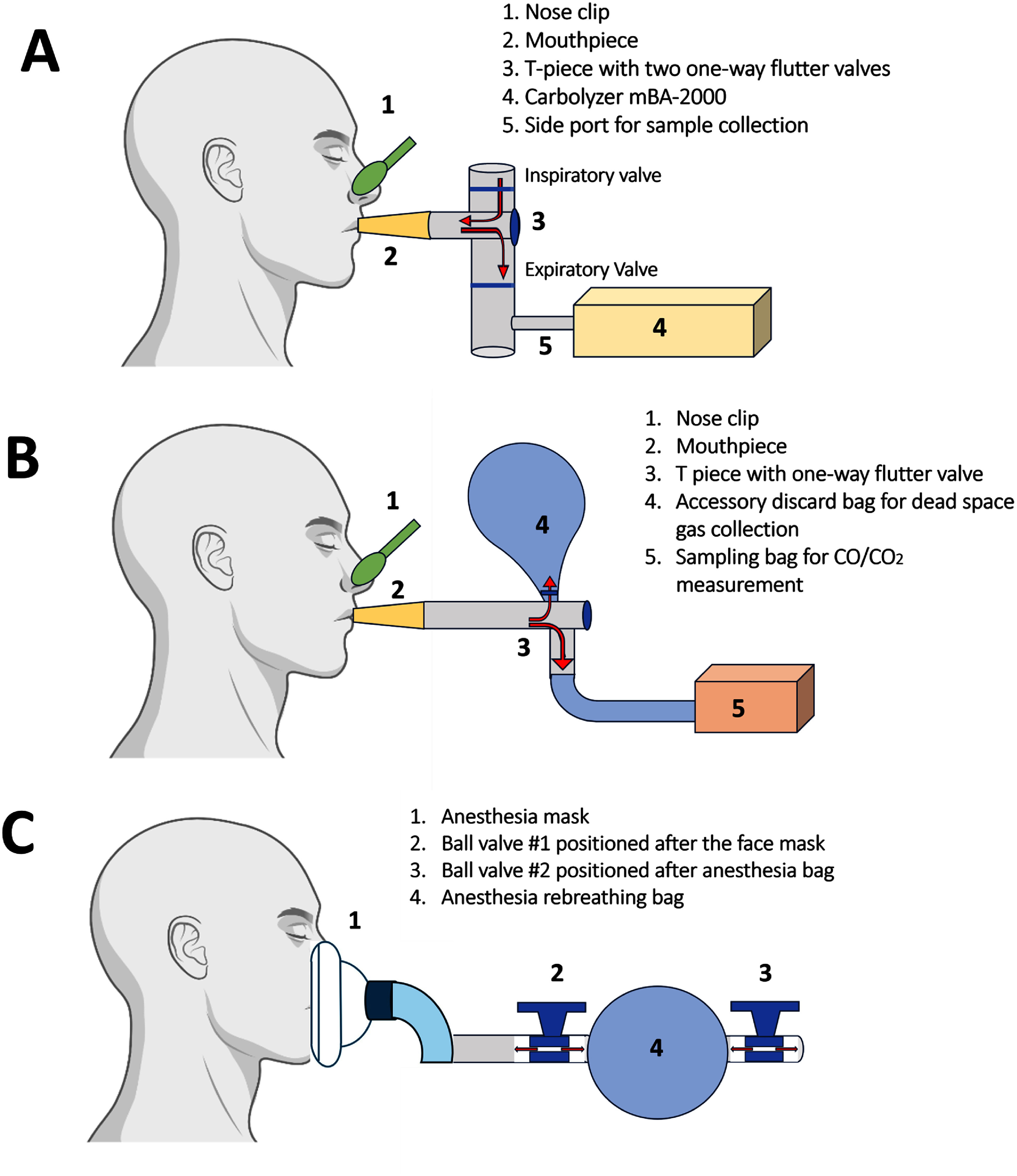
Schematics of the three eCO collection techniques. **Panel (A)** presents the **ConB** approach. A T piece with two one-way valves allows ambient room air to be inhaled but prevents the mixing of exhaled gas with ambient air, and a side port connects the collected gas directly to the Carbolyzer machine. **Panel (B)** depicts the **BrH** technique in which the exhaled gas first fills the accessory bag with dead-space gas, and then a valve opens, directing the alveolar expiratory gas to the sample bag. The sample bag was then connected to a Carbolyzer machine. **Panel (C)** shows the **SrB** technique, in which an anesthesia mask is placed over the face to achieve a tight seal around the nose and mouth. The participant breathes normally into the apparatus and takes and holds a deep breath from a mixed air reservoir. The gas was collected using a QuinTron Alveo Sampler breath collection kit.

With participants standing and at rest, maximal inspiration was performed, followed by breath-holding. An anesthesia mask was then placed over the face to ensure a tight seal around the nose and mouth. Valve 1 was opened, and the participant was instructed to breathe normally into the apparatus, while valve 2 remained closed, and the mask was sealed. After 30 s, the participants took and held a deep breath from the gas mixture inside the bag. A nose clip was applied, and a Quintron-alveolar sampler bag was filled with expiratory gas. Like the **BrH** method, CO/CO_2_ levels were measured using a Carbolyzer machine.

### 2.4. Statistical analysis

Demographic and clinical laboratory characteristics were summarized and compared among the groups. Mean (M), Coefficient of variation (CV) and standard deviation (SD) were used to quantify and compare the variation measurements. Student’s *t*-test was used to compare two groups. When more than two groups were compared, analysis of variance (ANOVA) for repeated measures, followed by Tukey’s *post hoc* test, was used. *P* $ \unicode{x2A7D} $ 0.05 was considered statistically significant.

## 3. Results

### 3.1. Inclusion/exclusion criteria, demographics, and screening laboratory tests

The inclusion and exclusion criteria are summarized in table 1. Particular emphasis was placed on excluding any known respiratory or hematologic conditions that might affect eCO and thorough documentation of smoking history and possible exposure to unusual levels of environmental CO. Demographic characteristics and the results of hematology screening parameters of the study participants in **Phase** I (10 subjects) and **Phase II** (30 subjects) are shown in table 2.

**Table 2.** Demographics and CBC data of participants enrolled in **Phase I** and **Phase II** of the study.

	Phase I			Phase II		
	(*n* = 10)			(*n* = 30)		
	Male (5)	Female (5)	Total (10)	Male (15)	Female (15)	Total (30)
Age (yr)	36.0 (15.5)	41.4 (12.6)	38.7 (13.6)	42.3 (16.3)	36.3 (14.5)	39.3 (15.4)
Height (cm)	181.0 (12.4)	165.2 (4.8)	173.3 (12.3)	174.3 (5.6)	163.0 (5.5)	168.7 (7.9)
Weight (kg)	105.4 (21.3)	81.0 (12.5)	93.2 (20.9)	89.0 (15.4)	76.1 (16.1)	82.6 (16.9)
Waist (cm)	99.2 (9.9)	90.4 (14.9)	94.8 (12.8)	97.1 (9.61)	85.6 (15.5)	91.3 (14.0)
BMI (kg m^−2^)	31.4 (3.5)	29.7 (4.2)	30.6 (3.7)	29.3 (5.0)	28.6 (5.7)	28.9 (5.3)
Hemoglobin (g/dl^−1^)	14.9 (1.0)	13.8 (1.4)	14.4 (1.3)	14.9 (.5)	13.0 (1.3)	13.9 (1.7)
Hematocrit (%)	43.8 (3.1)	41.6 (3.7)	42.7 (3.4)	42.9 (3.1)	39.1 (3.6)	41.0 (3.9)
RBC (10^9^ *μ*l^−1^)	5.0 (0.3)	4.7 (0.5)	4.8(0.4)	4.9 (0.5)	4.3 (0.4)	4.6 (0.5)
RDW (%) (normal range: 12–15)	13.12 (1.7)	14.5 (87)	13.8 (1.4)	13.6 (1.1)	13.3 (0.7)	13.4 (0.9)
Reticulocyte count (%) (normal range: 0.6–2.4)	1.5 (0.4)	1.1 (0.3)	1.3 (0.4)	1.4 (0.6)	1.4 (0.6)	1.4 (0.6)

RBC: red blood cell; RDW: red cell distribution width; Hemoglobin normal range: adult male: 13.5 0 17.5/ adult female: 12.0–16.0; RBC normal range: adult male: 4.5–6.0/adult female: 4.0−5.2; numbers represent means with ± 1SD in parentheses.

### 3.2. Phase I

#### 3.2.1. eCO measurements between 07:30 and 17:00 using BrH and ConB methods

**Figure 3.**
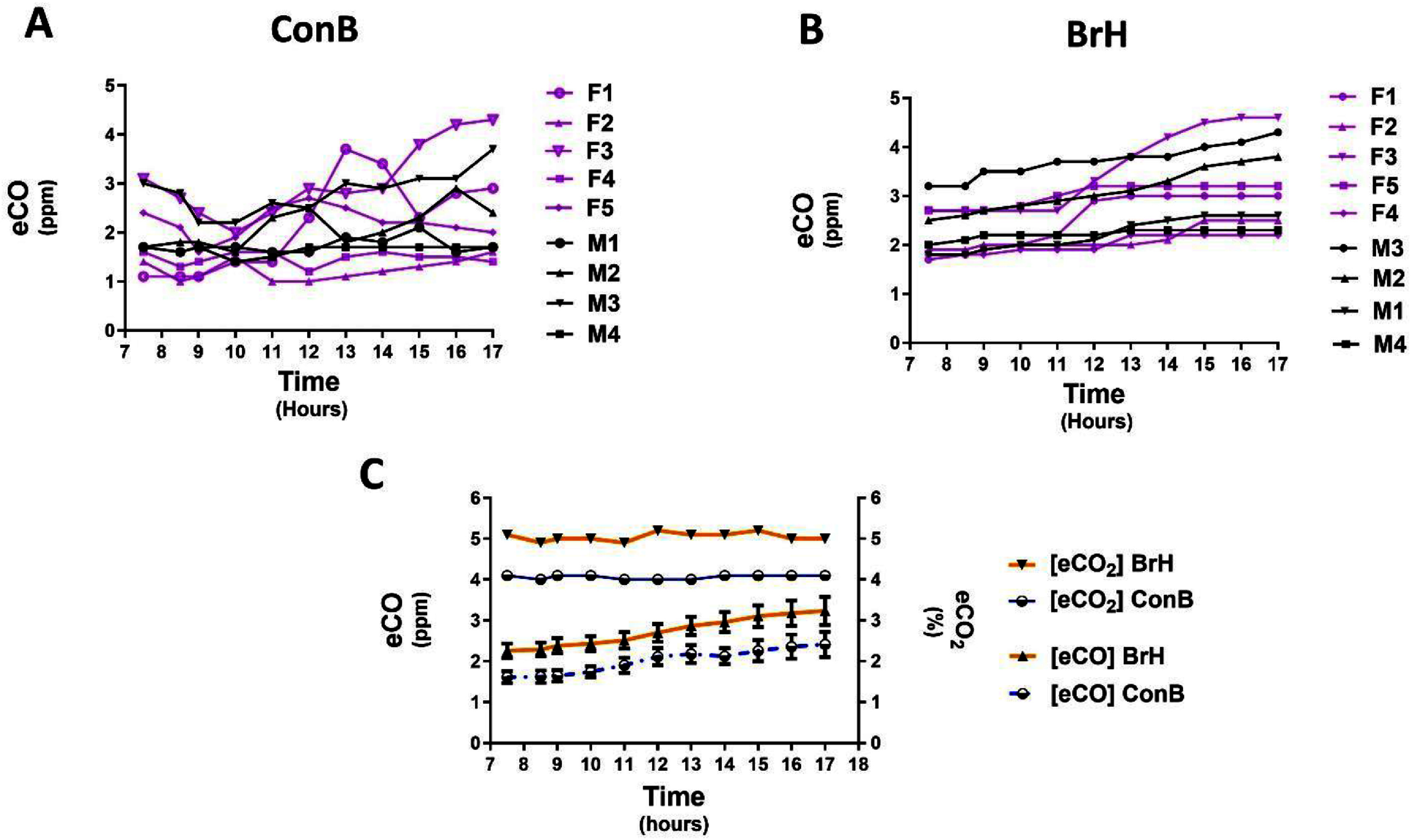
Phase I comparison of BrH and ConB techniques. **Panel (A)** eCO concentration variation using **ConB** method during a day (07:30–17:00) for nine subjects (**5F purple/4 M black**). **Panel (B)** eCO concentration variation using **BrH** method during a day (07:30–17:00) for nine subjects (**5F purple/4 M black**). **Panel (C)** Average eCO and eCO_2_ concentration variations between 07:30 and 17:30 using the two techniques. Breath-holding, **BrH**; Continuous breathing, **ConB**.

Figure 3**, Panels (A)** and **(B),** respectively, show the **ConB** and **BrH** eCO diurnal variation for the individual participants. Figure 3(**Panel C**) compares the diurnal profile of both techniques by averaging each time point and includes the simultaneous CO_2_ measurements. eCO, corrected for H_2_, appeared to be lower in the morning and increased gradually throughout the afternoon for both methods. Using repeated measures one-way ANOVA with Tukey’s *post hoc* correction, the **BrH** eCO concentration on average at 07:30 (2.3 ppm ± 0.5) was significantly different (*P* ⩽ 0.05) from all time points except at 09:00, 11:00, and 12:00. Pre-prandial and fasting H_2_ concentrations ranged from 3 to 8 ppm and, therefore, did not significantly affect the accuracy of eCO. However, postprandial H_2_ concentrations were as high as 17 ppm in some participants. CO_2_ was rarely equal to or higher than 4% using the **ConB** method. In contrast, the measured concentration of CO_2_ with the **BrH** technique consistently equaled or exceeded 4.6%, a level considered to reflect a valid ETCO_2_ sampling [33]. The correlation between the two methods was substantial but suggested room for improvement. (***R***^2^ = 0.86 (supplemental figure 1).

**Figure 4.**
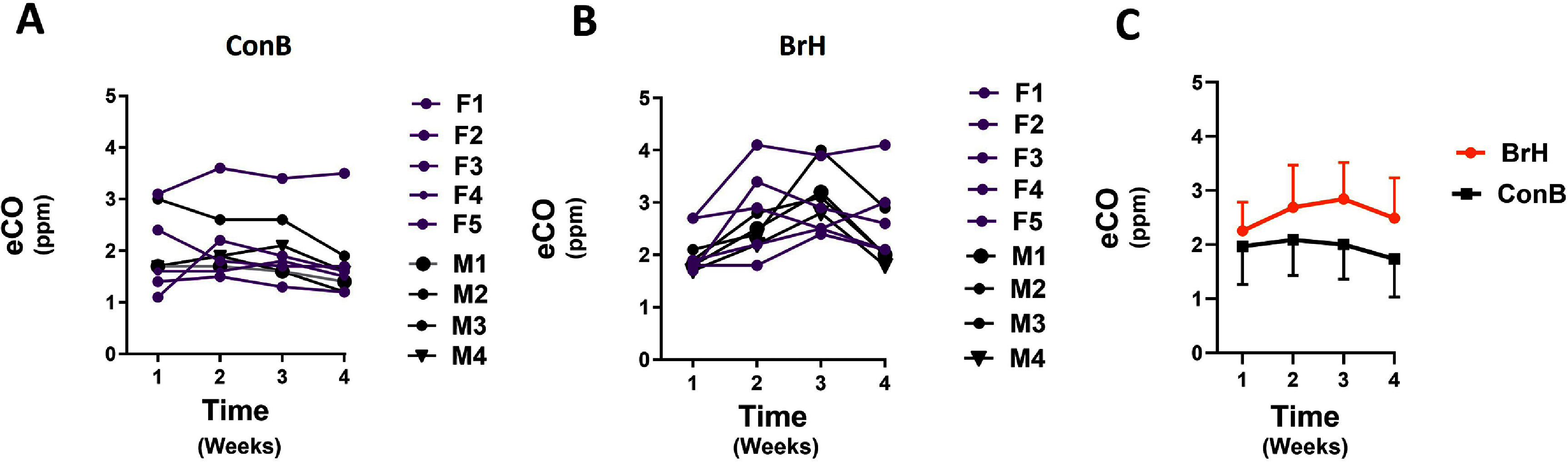
Phase I study weekly eCO weekly concentration variation using the BrH techniques during a day (08:00 until 10:00) for ten subjects. **Panel (A)** shows the individual participant profiles for **ConB** (**5F purple/5 M black**). **Panel (B)** demonstrates the individual participant profiles for **BrH** (**5F purple/5 M black) Panel (C)** depicts the average ± SD for **ConB** and **BrH**. Breath holding: **BrH**; Continuous breathing: **ConB**.

**Days 1, 8, 15, and 22: Weekly repeated eCO fasting measurements between 07:30 at 10:00 using BrH and ConB methods**. Following the day 1 diurnal variation assessment, the week-to-week variation was determined. Prebreakfast samples from 0800 to 1000 using the **ConB** (figure 4**, Panel (A)**) and **BrH (**figure 4**, Panel (B))** methods were collected. eCO levels varied from week to week for both methods. Figure 4**, Panel (C)** shows the average results for both methods. There was a statistically significant difference between weeks 1 and 2 with a standard *t*-test (*P* = 0.044) for the **BrH** method, but repeated measures one-way ANOVA with Tukey’s correction did not achieve statistical significance because of the high overall variance. Table 3 further summarizes the results of **Phase 1**. The CV was 10% or greater in all participants over the course of the 4-week testing.

**Table 3.** **Phase 1** study: fasting prebreakfast eCO (ppm) measured with **ConB** and **BrH** techniques every week for four weeks.

ConB									
	Female					Male			
Subjects	F1	F2	F3	F4	F5	M1	M2	M3	M4
WK1	1.1	1.4	3.1	1.6	2.4	1.7	1.7	3	1.7
WK2	2.2	1.5	3.6	1.6	1.8	1.7	1.9	2.6	1.9
WK 3	1.9	1.3	3.4	1.8	1.7	1.6	1.6	2.6	2.1
WK4	1.6	1.2	3.5	1.5	1.7	1.4	1.2	1.9	1.6
Mean	1.7	1.4	3.4	1.6	1.9	1.6	1.6	2.5	1.8
SD	0.5	0.1	0.2	0.1	0.3	0.1	0.3	0.5	0.2
CV	28%	10%	6%	8%	18%	9%	18%	18%	12%

BrH									

	Female					Male			
Subjects	F1	F2	F3	F4	F5	M1	M2	M3	M4

WK1	1.7	1.9	2.7	1.8	2.7	1.8	2.5	3.2	2
WK2	3.4	2.2	4.1	1.8	2.9	1.9	2.8	3.1	2
WK 3	2.9	2.5	3.9	2.4	2.5	2.1	2.4	4	2.9
WK4	2.6	2.1	4.1	2.1	3	1.7	2.2	2.8	1.8
Mean	2.7	2.2	3.7	2	2.8	1.9	2.5	3.3	2.2
SD	0.7	0.3	0.7	0.3	0.2	0.2	0.3	0.5	0.5
CV	27%	11%	18%	14%	8%	9%	10%	16%	23%

### 3.3. Phase II

#### 3.3.1. Phase II rationale

The diurnal variation observed was consistent with previous studies in the literature [27, 34], but the apparent week-to-week variability was unanticipated, in part because this aspect has not been explored comprehensively in the published literature to our knowledge [3, 18, 34]. This prompted a **Phase II** study to further assess week-to-week variation in a healthy population. In addition, the **ConB** approach was replaced by a third collection method, **SrB**. This technique is a simplified version (See **Methods**) of an approach demonstrated by other investigators to provide potentially more accurate alveolar air [34, 35]. **Phase II** was performed approximately 12 months after **Phase I. Phase II**, in addition to the week-to-week variability of the eCO, also included the evaluation of the reproducibility of the two techniques by performing repeated fasting samples from 08:00 to 10:00.

#### 3.3.2. Prebreakfast morning weekly eCO variation

**Figure 5.**
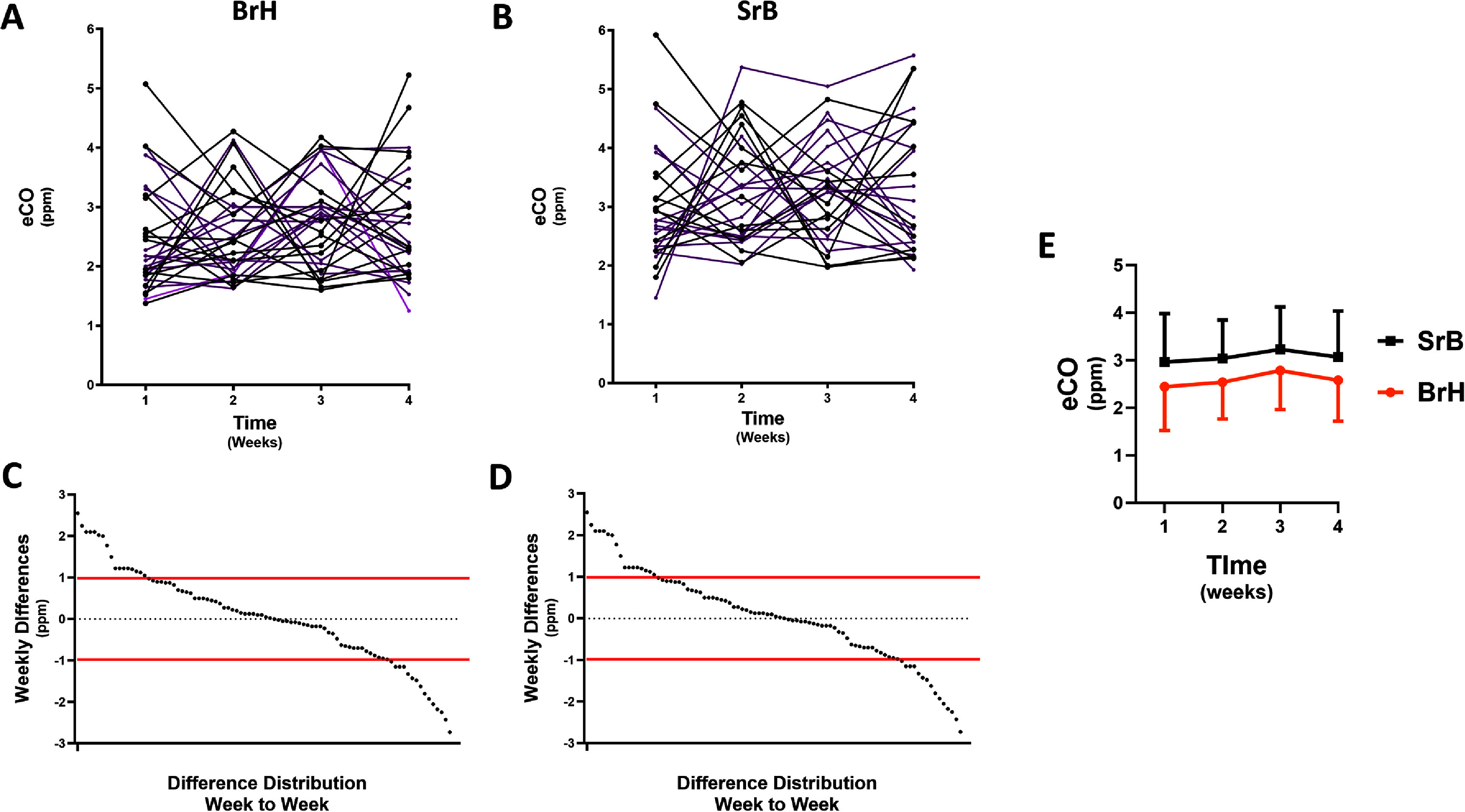
**Phase II** fasting prebreakfast weekly eCO variation using the BrH and SrB techniques. **Panels (A) and (B)** graphically show the **BrH** and **SrB** changes over time in all 30 subjects, demonstrating the variation. (**15 F purple/15 M black**) **Panels C** and **D** show the respective distribution of weekly differences for each of the thirty **Phase II** participants. **Panel E** depicts the mean ±SD. Breath holding: **BrH**. Short rebreathing: **SrB.**

Figure 5**, Panels (A)** and **(B),** show the **BrH** and **SrB** weekly changes in eCO for each of the 30 subjects. **Panels (C)** and **(D)** show the respective distribution of weekly differences for each the thirty **Phase II** participants calculated as follows: week 2 minus week 1, week 3 minus week 2, and week 3 minus week 3. The range of BrH differences was 0–2.7 ppm, 36% of the differences were ⩾ 1.0 ppm, and 13% were ⩾ 2 ppm. Similarly, for **SrB**, the range of differences was 0–2.95, 40% were ⩾ 2 ppm, and 11% ⩾ 2 ppm. **Panel E** depicts the average weekly values for the two methods. Although there appeared to be a trend towards an increase in the average values, this was not statistically significant.

**Figure 6.**
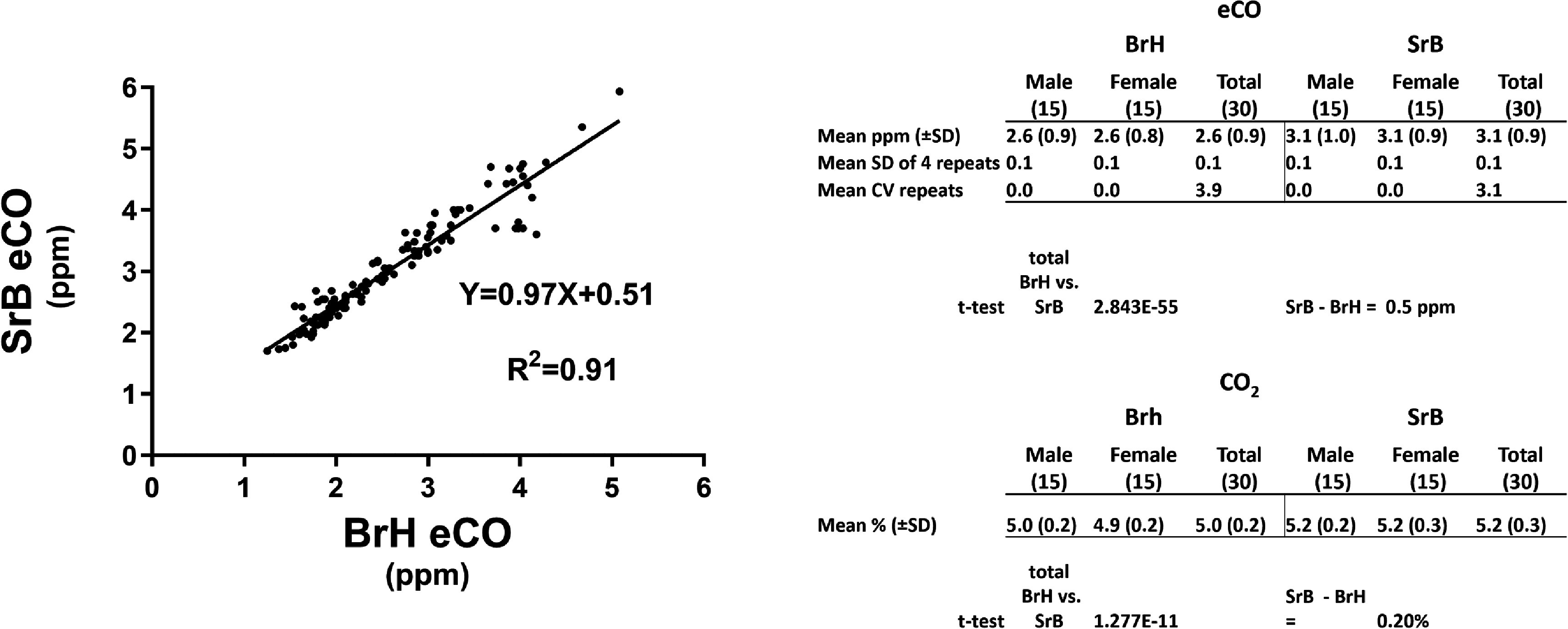
**Correlation analysis of SrB vs. BrH methods together with a tabular comparison.** The graph shows the results of a regression analysis relating the **SrB** and **BrH** methods in which all the fasting eCO samples for the four weeks of **Phase II** (120 **SrB** vs. 120 **BrH**) were included. The table summarizes some of the key quantitative comparisons for eCO and CO_2_. Coefficient of variation: **CV**. Breath-holding: **BrH**. Short rebreathing: **SrB.**

Figure 6 further compares the two techniques. Correlation analysis of the two methods showed a high ***R*^2^** value of 0.91. The tabular data summarizes the average eCO values for the **BrH** and **SrB** collection methods for the 4 weeks of **Phase II**. The **SrB** eCO results were consistently higher than **BrH** (0.5 ppm) and was highly significant. The SD for the eCO collections repeated four times in succession was low for both methods with an average CV of approximately 4%. There was no statistically significant difference between males and females. Importantly, both methods generated CO_2_ levels greater than 4.6% and the CO_2_ values by **SrB** method were significantly greater than the **BrH** (0.2%).

### 3.4. Discussion

This study aimed to examine factors that may cause variations in eCO, including the measurement protocol, diurnal changes, and week-to-week differences. Understanding these factors would allow for the development of standard protocols that minimize variation and enhance the application of eCO to study physiological and disease states. The study was performed in two **Phases**. In **Phase I**, two breathing protocols, **ConB** and **BrH,** were evaluated. While both methods gave consistent diurnal variation, the **ConB** technique was judged inadequate because it not only gave lower eCO values but also did not achieve CO_2_ concentrations reflecting sufficient alveolar sampling. In **Phase II** we compared **BrH** with a third technique, **SrB**, and evaluated both reproducibility and week-to-week changes. Both **BrH** and **SrB** produced CO_2_ concentrations that reached a threshold that was consistent with adequate alveolar sampling. For both methods, the eCO values were essentially identical within the method of repeated sampling over 20–30 min. However, eCO for **SrB** were consistently about 0.5 ppm higher than for **BrH**. Similarly, the CO_2_ measurements were approximately 0.2% higher. These results support the possibility of using breath-holding and/or short rebreathing methods to measure the value of exhaled CO that reflects the alveolar level and is reproducible over a 30-to-60 min time frame [17, 36, 37]. However, the eCO measurements for both **SrB** and **BrH** varied weekly in many participants. This variation was observed even though the studied population had been carefully screened for health conditions and environmental exposures known to affect circulating CO [10, 26, 34]. We conclude that factors distinct from collection methodology and environmental conditions within the CRU contribute to eCO and cause variation, which makes it difficult to interpret the contribution of modest changes in physiological and/or disease states.

Early methods using closed rebreathing circuits to assess CO production rate by measuring the linear increase in circulating CO were complex and not suited for routine measurements [15, 34]. In 1992, Strocchi *et al* [32] showed that eCO collected using a breath-holding method, in which the initial dead space of exhaled air was discarded, correlated well with a simultaneously performed ‘gold standard’ CO production rate measurements. CO_2_ was quantified to indicate adequate alveolar air collection. Other investigators have utilized airtight face masks and one-way valves to prevent mixing with ambient air [36] and factoring ventilation status [35] during sampling, while providing a collection that, based on CO_2_ concentrations, implied adequate collection. Alternative approaches include rebreathing strategies in which each inhalation consists of previously exhaled gases [28]. Through repeated cycles of inhalation and exhalation, eCO progressively increases to achieve maximal equilibrium with blood CO concentration and may represent a value higher than the actual ETCO [31].

In our study, the ranges of eCO values for both **BrH** and **SrB** (∼1–4 ppm) are consistent with concentrations published using similar methods in the smoking cessation literature [38, 39], hemolysis assessment in the newborn [18, 19, 40], and physiological variation in red cell lifespan calculations, as it may be related to diseases such as diabetes [25, 41–43] and inflammatory conditions often involving the pulmonary system [21, 44–47]. Additionally, consistent with prior studies, we detected significant hourly differences in eCO with an increase in eCO values throughout the day. Specifically, Coburn reported a variable CO production rate in one male patient tested for a 48 h period, peaking (∼ 3–4 fold greater than nadir) at around midnight on the second day [14]. Levitt *et al* [27], using a novel technique of measuring ETCO corrected for ambient CO, observed in seven healthy subjects a 26% increase in CO with a nadir at about midnight [2, 27]; in our study, we collected samples after a period of sitting at rest with stable vital signs to minimize the impact of acute changes in ventilation [26]. Finally, Sandberg *et al*, using a breath-holding approach and the Smokerlyzer Micro with a sensitivity of about one ppm and with less specificity than gas chromatography and infrared devices, performed a 1 h interval time course during the day on seven nonsmoker controls; they showed an increase at lunchtime, although they did not test for possible effects of H_2_ [20]. A consensus from the literature and our data strongly indicates that the time of day is a method-independent contributor to eCO and needs to be accounted for when interpreting the implications of a single eCO value.

To our knowledge, this study is the first to systematically assess week-to-week eCO variations. Other investigators have reported evidence of variation over days to weeks, but typically as a peripheral observation in a study focused on other aspects. For example, Yamaya *et al*, using a breath-holding technique, showed that eCO levels during an upper respiratory infection averaged ∼ 5.8 ppm but decreased to approximately 1.2 ppm three weeks after the resolution of symptoms [48]. A more recent study by Ye *et al*, which focused on comparing the variation of the Levitt method for eCO collection with ^15^N-glycine labeling technique for assessing red cell lifespan, showed a clear week-to-week variation with the eCO method [22]. In this small-scale study involving only five control subjects and three hemolysis patients, a typical participant had eighteen eCO measurements over the course of 180 d, approximately at week intervals coinciding with blood draws for ^15^N-heme analysis. The eCO results were in the 1–3 ppm range with a SD as high as 0.60, with corresponding lifespan calculations ranging from approximately 80 to 110 d.

Our results regarding diurnal and week-to-week variation using three different methods suggest that a one-time measurement of eCO may need to be interpreted with caution. These findings have implications for the application of eCO to assess normal physiology and disease states. For example, since the earliest CO production rate studies, there has been a focus on the estimation of red cell lifespan [49]. A formula for this was derived by Strocchi *et al* [32], which incorporated a number of significant assumptions. Since then, many authors have used the Strocchi formula to estimate hemoglobin turnover and red cell lifespan from eCO measured in a variety of ways [22]. Perhaps the most established clinical application for using eCO as a measure of hemolysis is in the setting of neonatal jaundice [8]. eCO is well-documented to have predictive value to assess the degree of pathologic hemolysis as an abnormally high eCO level can be distinguished from normal when subjected to receiver operating characteristic (ROC) curve analysis [50]. However, if the goal is to use eCO to determine the impact of normal variation of red cell lifespan on the relationship between blood glucose and HbA1c (which depends on both blood glucose and red cell lifespan [51]) and then adjust the final value to reflect the impact, this degree of week-to-week variation is unlikely to be informative. More specifically, applying the Strocchi equation with the assumption that eCO reflects the range of normal for red cell lifespan [52, 53], eCO would vary only from about 1.4–2.3 ppm. The current study demonstrating weekly variation often exceeds 1 ppm precludes applying eCO to adjust HbA1c for red cell life span

Our results suggest that improving the understanding of the physiology underlying CO production in normal and disease states will be a focus of future research. Efforts to refine techniques of collection and the sensitivity and specificity of measurement remain important. For example, Non-Dispersive Infrared sensors could be an option for measuring eCO due to their precision, non-invasive nature, and ability to provide real-time measurements [54]. A representative example that reflects these challenges is the interpretation of eCO and the need for further research is asthma. Most studies on asthma demonstrate an increased level of systemic and exhaled eCO, and the correlation between increased eCO and asthma is well-documented [55–57]. The increased eCO is thought to originate from the induction of airway epithelial cell HO as a part of the underlying inflammatory process [58]. However, how to apply this in a clinically useful manner is not clear. For example, a study by Ohara *et al* [21] assessed eCO in asthmatic infants and toddlers. The study showed that patients with an established diagnosis of asthma, eCO a cut-off ⩾ 2 ppm discriminated an active asthma episode from asymptomatic state with a sensitivity of 95.6%. However, the specificity was only 43.3%. Moreover, even with a cutoff level of ⩾ 3 ppm, eCO established the diagnosis of asthma with a sensitivity of only 38.9% and a specificity of 74.1%. Improving the value of eCO collection in asthma and other disease states is likely to be dependent on advances in understanding the precise role of HO, how it varies in individuals with the apparent similar underlying pathology, and how this affects eCO. The variation in eCO in our study, although presumably explained to some extent by differences in exhalation flow rate and other collection technique issues, may also be ultimately explained by poorly understood physiologic factors, including subclinical changes in HO.

Our study has several limitations. The first limitation is the specificity of the Carbolyzer instrument measurements. Although the instrument is sensitive and measures CO and CO_2_ in real time, electrochemical detector technology is not as specific as gold-standard methods, such as infrared spectroscopy [54] or gas chromatography. However, we accounted for the lack of specificity at high H_2_ concentrations by subtracting these values. Regardless, this adds some possibility of additional inaccuracy to the final numbers. The second is the degree to which our eCO levels reflect ETCO. Samples that exceeded the 4.6% threshold of eCO_2_ were considered adequate alveolar samples, but this may not have been a valid assumption. Third, although participants were screened for environmental factors that might influence eCO, and the Carbolyzer eCO was corrected for ambient CO concentrations, it is possible that environmental factors, particularly distinct from the controlled environment of our CRU, may have significantly contributed to the results. Fourth, our approach could have been enhanced by accounting for the impact of factors such as the expiratory flow rate, as summarized in a recent comprehensive study by Ghorbani *et al* [26] Finally, the difference in the absolute values of the **BrH** and **SrB** techniques raises a question about which reflects the true alveolar concentration of CO. The difference is relatively small but is consistently observed in most of the samples obtained. Moreover, because the CO_2_ concentrations were also significantly higher with the **SrB** technique, our conclusion, within the limitations cited, is that the **SrB** approach is likely to reflect ETCO better than **BrH**. (**see** table 4 **summarizing the three methods**) In addition, though not specifically surveyed, it was anecdotally observed that the **SrB** method, similar to reports by other investigators [28] applying similar techniques, was noted to be easier for participants to complete.

**Table 4.** Comparison of the eCO collection methods.

Parameter	Continuous breathing	Breath holding	Short rebreathing
Average fasting eCO (ppm)	∼ 1.9	∼ 2.6	∼ 3.1
Average fasting CO2 (%)	∼ 4.1	∼ 5	∼ 5.2
Consistency/reproducibility (CV)	∼ 9%	∼ 4%–5%	∼ 4%–5%
Comfort for subject	Good	Can be a challenge to hold breath	Good
Ease of Execution	Good	acceptable	Requires supplemental Oxygen source
eCO measurement	Continuous	One sample	One sample

## 4. Conclusions

This study examined the collection of eCO using three techniques with an electrochemical detector. Given the consistent minute-to-minute concentrations and careful screening of the study participants, the diurnal and week-to-week variations in eCO levels were unlikely to be due to technical issues of measurement and collection. Therefore, we conclude that there is a significant physiological variation in the production of CO within the body, a phenomenon that is not yet fully understood. This presents a limitation in using CO as a reliable marker for assessing the physiological and disease states linked to changes in ETCO.

## Data Availability

The data cannot be made publicly available upon publication because the cost of preparing, depositing and hosting would be prohibitive with the terms of this research project. The data supporting the findings of this study are available upon reasonable request from the authors.

## References

[1] Wallace M A G, Kormos T M, Pleil J D (2016). Blood-borne biomarkers and bioindicators for linking exposure to health effects in environmental health science. J. Toxicol. Environ. Health B.

[2] Ryter S W (2010). Special issue on carbon monoxide and exhaled biomarkers in human disease. J. Breath Res..

[3] Ryter S W, Choi A M K (2013). Carbon monoxide in exhaled breath testing and therapeutics. J. Breath Res..

[4] Ryter S W, Alam J, Choi A M K (2006). Heme oxygenase-1/carbon monoxide: from basic science to therapeutic applications. Physiol. Rev..

[5] Christensen R D, Yaish H M (2016). Hemolysis in preterm neonates. Clin. Perinatol..

[6] Bensinger T A, Gillette P N (1974). Hemolysis in sickle cell disease. Arch. Intern. Med..

[7] Kang L-L, Ma Y-J, Zhang H-D (2020). Carbon monoxide breath test assessment of mild hemolysis in Gilbert’s syndrome. Medicine.

[8] Christensen R D, Malleske D T, Lambert D K, Baer V L, Prchal J T, Denson L E, Gerday E, Weaver Lewis K A, Shepherd J G (2015). Measuring end-tidal carbon monoxide of jaundiced neonates in the birth hospital to identify those with hemolysis. Neonatology.

[9] Coburn R F (1970). Endogenous carbon monoxide production. New Engl. J. Med..

[10] Ryter S W, Otterbein L E (2004). Carbon monoxide in biology and medicine. Bioessays.

[11] Cunnington A J, Hormbrey P (2002). Breath analysis to detect recent exposure to carbon monoxide. Postgrad. Med. J..

[12] Ryter S W, Kim H P, Nakahira K, Zuckerbraun B S, Morse D, Choi A M K (2007). Protective functions of heme oxygenase-1 and carbon monoxide in the respiratory system. Antioxid. Redox Signal.

[13] Ryter S W (2021). Significance of Heme and Heme degradation in the pathogenesis of acute lung and inflammatory disorders. Int. J. Mol. Sci..

[14] Coburn R F, Forster R E, Kane P B (1965). Considerations of the physiological variables that determine the blood carboxyhemoglobin concentration in man. J. Clin. Invest..

[15] Coburn R F, Blakemore W S, Forster R E (1963). Endogenous carbon monoxide production in man. J. Clin. Invest..

[16] Virtue M A, Furne J K, Ho S B, Levitt M D (2004). Use of alveolar carbon monoxide to measure the effect of ribavirin on red blood cell survival. Am. J. Hematol..

[17] Furne J K, Springfield J R, Ho S B, Levitt M D (2003). Simplification of the end-alveolar carbon monoxide technique to assess erythrocyte survival. J. Lab. Clin. Med..

[18] Tidmarsh G F, Wong R J, Stevenson D K (2014). End-tidal carbon monoxide and hemolysis. J. Perinatol..

[19] Bahr T M, Shakib J H, Stipelman C H, Kawamoto K, Lauer S, Christensen R D (2021). Improvement initiative: end-tidal carbon monoxide measurement in newborns receiving phototherapy. J. Pediatr..

[20] Sandberg A, Sköld C M, Grunewald J, Eklund A, Wheelock Å M (2011). Assessing recent smoking status by measuring exhaled carbon monoxide levels. PLoS One.

[21] Ohara Y, Ohara T, Hashimoto K, Hosoya M (2020). Exhaled carbon monoxide levels in infants and toddlers with episodic asthma. Fukushima J. Med. Sci..

[22] Ye L (2021). Comparison of Levitt’s CO breath test and the 15N-glycine labeling technique for measuring the lifespan of human red blood cells. Am. J. Hematol..

[23] Medina A, Ellis C, Levitt M D (1994). Use of alveolar carbon monoxide measurements to assess red blood cell survival in hemodialysis patients. Am. J. Hematol..

[24] Ryter S W (2021). Heme Oxgenase-1, a cardinal modulator of regulated cell death and inflammation. Cells.

[25] Virtue M A, Furne J K, Nuttall F Q, Levitt M D (2004). Relationship between GHb concentration and erythrocyte survival determined from breath carbon monoxide concentration. Diabetes Care.

[26] Ghorbani R, Blomberg A, Schmidt F M (2020). Impact of breath sampling on exhaled carbon monoxide. J. Breath Res..

[27] Levitt M D, Ellis C, Levitt D G (1994). Diurnal rhythm of heme turnover assessed by breath carbon monoxide concentration measurements. J. Lab. Clin. Med..

[28] Garvican L A, Burge C M, Cox A J, Clark S A, Martin D T, Gore C J (2010). Carbon monoxide uptake kinetics of arterial, venous and capillary blood during CO rebreathing. Exp. Physiol..

[29] Khasag N (2013). Monitoring of exhaled carbon monoxide and carbon dioxide during lung cancer operation. Eur. J. Cardio-Thoracic Surg..

[30] Messina Z, Patrick H (2022). Partial pressure of carbon dioxide. StatPearls.

[31] Lawal O, Ahmed W M, Nijsen T M E, Goodacre R, Fowler S J (2017). Exhaled breath analysis: a review of “breath-taking” methods for off-line analysis. Metabolomics.

[32] Strocchi A, Schwartz S, Ellefson M, Engel R R, Medina A, Levitt M D (1992). A simple carbon monoxide breath test to estimate erythrocyte turnover. J. Lab. Clin. Med..

[33] Kodali B S (2013). Capnography outside the operating room. Anesthesiology.

[34] Coburn R F (2012). The measurement of endogenous carbon monoxide production. J. Appl. Physiol..

[35] Cavaliere F, Volpe C, Gargaruti R, Poscia A, Di Donato M, Grieco G, Moscato U (2009). Effects of acute hypoventilation and hyperventilation on exhaled carbon monoxide measurement in healthy volunteers. BMC Pulm. Med..

[36] Mitlyng B L, Singh J A, Furne J K, Ruddy J, Levitt M D (2006). Use of breath carbon monoxide measurements to assess erythrocyte survival in subjets with chronic diseases. Am. J. Hematol..

[37] Christensen R D, Lambert D K, Henry E, Yaish H M, Prchal J T (2014). End-tidal carbon monoxide as an indicator of the hemolytic rate. Blood Cells Mol. Dis..

[38] Wald N J, Idle M, Boreham J, Bailey A (1981). Carbon monoxide in breath in relation to smoking and carboxyhaemoglobin levels. Thorax.

[39] Kirkham A J, Guyatt A R, Cumming G (1988). Acute effect of smoking on rebreathing carbon monoxide, breath-hold carbon monoxide and alveolar oxygen. Clin. Sci..

[40] Maisels M J, Pathak A, Nelson N M, Nathan D G, Smith C A (1971). Endogenous production of carbon monoxide in normal and erythroblastotic newborn infants. J. Clin. Invest..

[41] Zhou S, Dong R, Wang J, Zhang L, Yu B, Shao X, Bai P, Zhang R, Ma Y, Yu P (2022). Red blood cell lifespan < 74 days can clinically reduce Hb1Ac levels in type 2 diabetes. J. Pers. Med..

[42] Huang Z, Liu Y, Mao Y, Chen W, Xiao Z, Yu Y (2018). Relationship between glycated haemoglobin concentration and erythrocyte survival in type 2 diabetes mellitus determined by a modified carbon monoxide breath test. J. Breath Res..

[43] Wang J, Zhang L, Bai Y, Wang X, Wang W, Li J, Zhou S (2022). The influence of shorter red blood cell lifespan on the rate of HbA1c target achieved in type 2 diabetes patients with a HbA1c detection value lower than 7. J. Diabetes.

[44] Slebos D-J, Ryter S W, Choi A M K (2003). Heme oxygenase-1 and carbon monoxide in pulmonary medicine. Respir. Res..

[45] Ohara Y, Ohrui T, Morikawa T, He M, Yasuda H, Yamaya M, Sasaki H (2006). Exhaled carbon monoxide levels in school-age children with episodic asthma. Pediatr. Pulmonol..

[46] Ryter S W, Ma K C, Choi A M K (2017). Carbon monoxide in lung cell physiology and disease. Am. J. Physiol. Cell Physiol..

[47] Jesenak M, Banovcin P, Havlicekova Z, Dobrota D, Babusikova E (2014). Factors influencing the levels of exhaled carbon monoxide in asthmatic children. J. Asthma.

[48] Yamaya M, Sekizawa K, Ishizuka S, Monma M, Mizuta K, Sasaki H (1998). Increased carbon monoxide in exhaled air of subjects with upper respiratory tract infections. Am. J. Respir. Crit. Care Med..

[49] Franco R S (2009). The measurement and importance of red cell survival. Am. J. Hematol..

[50] Christensen R D, Bahr T M, Wong R J, Vreman H J, Bhutani V K, Stevenson D K (2023). A “gold standard” test for diagnosing and quantifying hemolysis in neonates and infants. J. Perinatol..

[51] Smith E P, Cohen R M (2015). Physiologic concepts that may revise the interpretation and implications of HbA1C in clinical medicine: an American perspective. J. Diabetes Sci. Technol..

[52] Khera P K (2015). Use of an oral stable isotope label to confirm variation in red blood cell mean age that influences HbA1c interpretation. Am. J. Hematol..

[53] Cohen R M, Franco R S, Khera P K, Smith E P, Lindsell C J, Ciraolo P J, Palascak M B, Clinton H J (2008). Red cell life span heterogeneity in hematologically normal people is sufficient to alter HbA1c. Blood.

[54] Fritsch T, Hering P, Mürtz M (2007). Infrared laser spectroscopy for online recording of exhaled carbon monoxide-a progress report. J. Breath Res..

[55] Yamaya M, Sekizawa K, Ishizuka S, Monma M, Sasaki H (1999). Exhaled carbon monoxide levels during treatment of acute asthma. Eur. Respir. J..

[56] Xie Z, Chai M, Gu W, Yuan H (2020). Changes in fractional exhaled nitric oxide, exhaled carbon monoxide and pulmonary function during the acute attack, treatment and remission phases of pediatric asthma. Transl. Pediatr..

[57] Zhang J, Yao X, Yu R, Bai J, Sun Y, Huang M, Adcock I M, Barnes P J (2010). Exhaled carbon monoxide in asthmatics: a meta-analysis. Respir. Res..

[58] Xia Z, Zhong W (2022). Immune regulation of heme oxygenase-1 in allergic airway inflammation. Antioxidants.

